# Individual Specialization to Non-Optimal Hosts in a Polyphagous Marine Invertebrate Herbivore

**DOI:** 10.1371/journal.pone.0102752

**Published:** 2014-08-04

**Authors:** Finn A. Baumgartner, Henrik Pavia, Gunilla B. Toth

**Affiliations:** Department of Biological and Environmental Sciences-Tjärnö, University of Gothenburg, Strömstad, Sweden; CSIR- National institute of oceanography, India

## Abstract

Factors determining the degree of dietary generalism versus specialism are central in ecology. Species that are generalists at the population level may in fact be composed of specialized individuals. The optimal diet theory assumes that individuals choose diets that maximize fitness, and individual specialization may occur if individuals' ability to locate, recognize, and handle different food types differ. We investigate if individuals of the marine herbivorous slug *Elysia viridis*, which co-occur at different densities on several green macroalgal species in the field, are specialized to different algal hosts. Individual slugs were collected from three original algal host species (*Cladophora sericea, Cladophora rupestris* and *Codium fragile*) in the field, and short-term habitat choice and consumption, as well as long-term growth (proxy for fitness), on four algal diet species (the original algal host species and *Chaetomorpha melagonium*) were studied in laboratory experiments. Nutritional (protein, nitrogen, and carbon content) and morphological (dry weight, and cell/utricle volume) algal traits were also measured to investigate if they correlated with the growth value of the different algal diets. *E. viridis* individuals tended to choose and consume algal species that were similar to their original algal host. Long-term growth of *E. viridis*, however, was mostly independent of original algal host, as all individuals reached a larger size on the non-host *C. melagonium*. *E. viridis* growth was positively correlated to algal cell/utricle volume but not to any of the other measured algal traits. Because *E. viridis* feeds by piercing individual algal cells, the results indicate that slugs may receive more cytoplasm, and thus more energy per unit time, on algal species with large cells/utricles. We conclude that *E. viridis* individuals are specialized on different hosts, but host choice in natural *E. viridis* populations is not determined by the energetic value of seaweed diets as predicted by the ODT.

## Introduction

Species vary greatly in resource use and factors that determine the degree of dietary generalism (polyphagy) and specialism (monophagy) among and within species are central in ecology because they affect the trophic transfer of energy through food webs [1], drive the evolution of ecological interactions [2], and maintain biodiversity [3]. Recently, however, the existence of true generalists has been questioned [4], and it has become clear that individuals within a polyphagous species or population can vary in their resource use (i.e. individual specialization) [5], [6]. One approach to understanding why a population of conspecific individuals with access to the same set of resources chooses to exploit them differently is via the framework of optimal diet theory (ODT) [7]. The ODT assumes that an individual is able to rank food items after the rate of energy assimilation per unit time, and select a diet that maximizes some currency related to fitness [7]. If sympatric conspecifics differ in their ability to locate, recognize, and handle different food types this may result in individual specialization [8].

Individual specialization was previously thought to be rare or have minimal effects on ecological processes [9], and was also ignored in many ecological studies. However, individual specialization is not uncommon and is an important contributor to processes such as polymorphism and speciation [5]. Furthermore, a recent review suggested that individual specialization tends to be common in upper trophic positions [6], but whether this reflects a true pattern or a sampling bias is not clear, as studies on individual specialization in herbivores (especially on small terrestrial insect and marine invertebrate herbivores) are relatively few. Many terrestrial insect herbivore species are specialized to one or a few plant species belonging to the same family [10], which may explain the fact that they are not frequently used as models to study individual specialization (but see [5]). Marine herbivores, on the other hand could be excellent models to study individual specialization at lower trophic levels, because, in contrast to terrestrial insects, most (but not all) marine herbivore species are considered generalists that feed on a broad variety of algal hosts [11].

The overall aim of the present study was to investigate if individuals of the marine herbivore *Elysia viridis* are specialized (*sensu* [5]) to different species of algae within the qualitative framework of the ODT. *E. viridis* belongs to a group of small marine invertebrate herbivores (sacoglossan opistobranch molluscs, or sea slugs) that uses the algal host plants both as habitat and food. One defining characteristic of the group is the possession of a highly specialized feeding apparatus that is used to pierce and suck cytoplasm from algal cells. In addition, sacoglossans have a number of other functional specializations in relation to their food algae including crypsis in morphology and coloration [12], [13], sequestration or synthesis of natural products for defense [12], [14]–[17], and sequestration of functional chloroplasts (kleptoplasty, *sensu* [18]) as a supplementary energy source through photosynthesis (e.g. [19]–[22]).

Sacoglossans in general are considered to be rather specialized marine herbivores and many species only use one or a few algae species as host plants. *E. viridis*, however, occurs sympatrically on several green macroalgal species including the siphonous/coenocytic green algal genera *Codium* and *Bryopsis*, the filamentous/septate green algal genera *Chaetomorpha* and *Cladophora*, and the filamentous red algal genus *Griffithisia* [23]–[25]. In the study area (Swedish west coast), the abundance and size distribution of *E. viridis* varies significantly between different green algal host species [26]. The highest number of small *E. viridis* are found on *Cladophora sericea*, intermediate numbers of significantly larger *E. viridis* are found on *Cladophora rupestris*, while fewer, intermediate sized animals are found on *Codium fragile*. *Chaetomorpha melagonium* and *Bryopsis sp.* are not inhabited by *E. viridis* in natural slug populations on the Swedish west coast [26].

We collected *E. viridis* from three algal host species in the field and compared short-term choice and consumption, as well as long-term growth (a proxy for fitness), of slugs on four different algal diets in the laboratory. We specifically hypothesized that if *E. viridis* individuals are specialized to live on, and feed from, different algal hosts, slugs will 1) associate with their original algal host species when given a choice, and 2) have a higher consumption on their original algal host species in the laboratory. Furthermore, if *E. viridis* individuals are able to rank and choose food items in terms of their fitness value as predicted by the ODT, slugs collected from different algal hosts in natural field populations will 3) show the highest growth rate on the original algal host species. Furthermore, we measured several nutritional (tissue protein (P), nitrogen (N), carbon (C) content, as well as the C:N ratio) and morphological (percent dry weight and cell volume) algal traits in order to determine if they correlated with the fitness value of the different algal diet species.

## Materials and Methods

### Collection and maintenance of organisms

*E. viridis* were collected at <5 m depth from three different species of green macroalgal hosts (*C. fragile*, *C. rupestris* and *C. sericea*, henceforth termed “original algal hosts”) in late July/early August 2010 or 2011 from two sites in the Koster Fjord, Sweden (Yttre Vattenholmen 58° 52′ 33.5″ N, 11°6′ 22.9″ E and Saltö Lyngnholmen 58° 51′ 45.3″ N, 11° 7′ 52.8″ E). No specific permissions were required to sample organisms at these locations and the study did not involve any endangered or protected species. *E. viridis* from the two sites were pooled and maintained in outdoor aquaria with running surface seawater (15.9±0.4°C, 23.9±0.3‰, mean ± SEM) and their original algal host as food for at least one month prior to their use in experiments. To our knowledge, the movement of *E. viridis* between different algal hosts in the field is not known. A recent analysis of the pigment composition of *E. viridis* populations in Portugal suggests that individuals fed only on the algal species from which they were collected [27]. However, Händeler and co-workers [28] detected two different algal species (likely two *Bryopsis* spp.), using molecular methods within a single individual *E. viridis* collected from France. While this indicates the possibility for movement between algal hosts, *E. viridis* in our study area are rather small and the tested algal species commonly do not grow in close connection. Therefore, we believe that the possibility of movement between different algal species in the field should be low for Swedish *E. viridis*, and that confining the slugs to a host for one month is representative of field conditions. Mortality was very low and selection should not have affected the outcome of the following experiments.

In order to acclimatize the *E. viridis* to indoor conditions, slugs were transferred to indoor aquaria at least two days prior to commencement of an experiment. In addition, separate individuals of four species of green macroalgae (*C. fragile, C. rupestris, C. sericea*, and the non-host *C. melagonium*, henceforth termed “algal diet species”) were collected from the same sites as *E. viridis* 1 to 10 days prior to use in experiments, and maintained in indoor aquaria with running seawater. The day before an experiment, tips from individual *C. fragile* thalli were cut, placed in containers and left in running seawater overnight to heal. This was done to reduce any weight changes associated with leakage due to the siphonous morphology of *C. fragile* and to ensure that only healthy pieces of the alga were used.

### *Elysia viridis* preference for different algal diet species

The preference of large (70±1 mg, mean ± SEM) and small (16±0.5 mg, mean ± SEM) *E. viridis* individuals from the three original algal hosts were assessed through multiple-choice assays using the four algal diet species in October 2010 and August 2011 respectively. *E. viridis* individuals that had remained feeding on their original algal hosts for either 3 (large animals) or 1 (small animals) month were simultaneously offered a choice of fresh pieces of the four algal diet species in individual square-based (9×9 cm, total volume 1 L) aquaria filled with 400 ml of seawater (n = 20). Algal pieces were randomly cable tied to the edges of the aquaria and one *E. viridis* was positioned in the center. The position of each *E. viridis* individual was observed every 2–4 h over a 40 h period (14 observations per replicate). The *E. viridis* preference was calculated by dividing the number of times an *E. viridis* individual was observed on a particular algal species by the total number of observations to yield percent host use.

Non-parametric Friedman's tests were used to analyze the diet preference of large and small *E. viridis* from different original algal hosts, based on the mean number of times (scores) individuals were observed on each algal species. Prior to analysis, scores were rank-transformed within each replicate assay [29]. Observations where an individual *E. viridis* was not positioned on an algae (no choice) were not included in analyses. Furthermore, replicates where *E. viridis* were observed on an alga less than 50% of the time (i.e. <7 observations) were excluded from the statistical analysis. Friedman's post hoc comparisons (Friedman's PHC) were used to determine the pairwise differences between mean scores on each algal species by *E. viridis* from each host and size class [29].

### *Elysia viridis* consumption of different algal diet species

The consumption by *E. viridis* collected from the three original algal hosts was assessed through no-choice feeding assays with new large (55±0.8 mg, mean ± SEM) and small (16±0.3 mg, mean ± SEM) slugs using the four algal diet species. Due to the difference in morphology of the algal diets (siphonaceous versus septate), *E. viridis* consumption was assessed by measuring the mass change of *C. fragile*, and by recording the number of damaged cells of *C. melagonium*, *C. sericea*, and *C. rupestris*. *E. viridis* individuals from each original algal host were placed individually in aquaria (0.2 L) with fresh pieces of one of the four algal diets (n = 10). In order to control for autogenic changes not associated with feeding, genetically identical pieces of algal diets without *E. viridis* were prepared in separate aquaria. The mass or number of damaged cells was determined prior to commencing the assay and when >50% of the diet algal piece appeared damaged or after a maximum of 96 h. Consumption rate of each individual *E. viridis* was calculated using the following equations:

Consumption rate (mg d^−1^)  =  
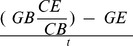
 for *C. fragile*,

Consumption rate (cells d^−1^)  =  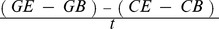 for *C. melagonium, C. rupestris*, and *C. sericea*, where GB and CB represent the grazed and control algal diets before, and GE and CE represent the grazed and control algal diets at the end of the experiment. t refers to time in days of the experimental run. Data were statistically analyzed separately for large and small *E. viridis* consuming different algal diet species using one-way ANOVAs with original algal host as a fixed factor.

### *Elysia viridis* growth on different algal diet species

The growth of *E. viridis* collected from the three original algal hosts was assessed through long-term no-choice feeding assays with the four algal diet species. 100 small *E. viridis* individuals from each original algal host were patted dry, weighed (16±0.2 mg), placed in separate seawater-filled aquaria (0.2 L), and provided with one of the four algal diet species or starved for 28 d (n = 20). Seawater was exchanged daily and animals were provided fresh algae every 3–4 d. Animals were kept under a 12∶12 h light:dark cycle with surface photosynthetically active radiation (PAR), seawater temperature, and salinity ranging from 5.0–8.1 µmol photons.m^−2^.s^−1^, 17.0–19.5°C, and 20–26‰ respectively during the experiment. Light was deliberately kept low in order to minimize growth of slugs due to photosyntetically active kleptoplasts. *E. viridis* growth was calculated by subtracting the initial mass from the final mass. Data on growth were statistically analyzed in a two-way ANOVA with original algal host and algal diet species as fixed orthogonal factors. Data on weight changes in starved animals were not included in the statistical analysis due to the small variances associated with their means.

### Nutritional and morphological traits of algal diet species

In order to assess if nutritional or morphological traits of the algal diets were correlated to the growth of *E. viridis*, we measured the percent dry mass, tissue N, C, and P content, and calculated the C:N ratio in each algal diet species each week of the experiment (n = 5). Algal pieces were blotted dry to remove excess water, weighed (wet weight), freeze-dried and reweighed (dry weight) and percent dry mass was calculated. Tissue N and C content of each sample was measured by combustion using a Fisons EA1108 CHNS-O element analyser. Tissue P content was measured colourimetrically using a modified version of Bradford's method [30]. Each sample was ground in 1 ml of 0.11 mol.ml^−1^ NaOH for 15 min using a Ball Mill (Retsch MM301) and was left to incubate in the fridge (4°C) for 22 h. After incubation each sample was centrifuged and 50 µl of the supernatant was mixed with 2.5 ml of the protein-binding reagent Coomassie Blue-G250 (Thermo Scientific). Samples were left to stand for 16 min to allow colour generation before measuring their absorbance at 595 nm on an UV/Vis spectrophotometer (Perkin Elmer Lambda XLS+). Absorbance values were converted to µg protein using calibration curves derived from a bovine serum albumin standard (Thermo Scientific). Although this method does not provide the absolute amount of soluble protein it is considered a reliable measure to compare soluble protein content between algal species [31]. Furthermore, the diameter and length of 10 randomly selected cells/utricles were measured in each algal diet species collected in weeks 2–4 of the experiment (n = 5) with an inverted microscope (Olympus IX71) equipped with an ocular micrometer, and the cell/utricle volume was calculated using the formula for the volume of a cylinder.

Data on the nutritional and morphological traits of the algal diets were statistically analysed in two-way ANOVAs with algal diet species and week collected as the fixed orthogonal factors. Data on cell/utricle volume were analysed in a three-way mixed-model nested ANOVA with algal diet species and week collected as fixed, orthogonal factors and algal individual as random factor nested within the interaction between the fixed factors. Means were compared using the Ryan, Einot, Gabriel and Welsh *F* (R-E-G-W *F*) procedure. Prior to ANOVAs, data were tested for homogeneity of variances with Levene's test, and data were log transformed when required. Simple linear regressions were used to analyse if mean growth of *E. viridis* on the different algal diet species correlated to any of the measured nutritional or morphological algal traits.

## Results

### *Elysia viridis* preference for different algal diet species

**Figure 1:**
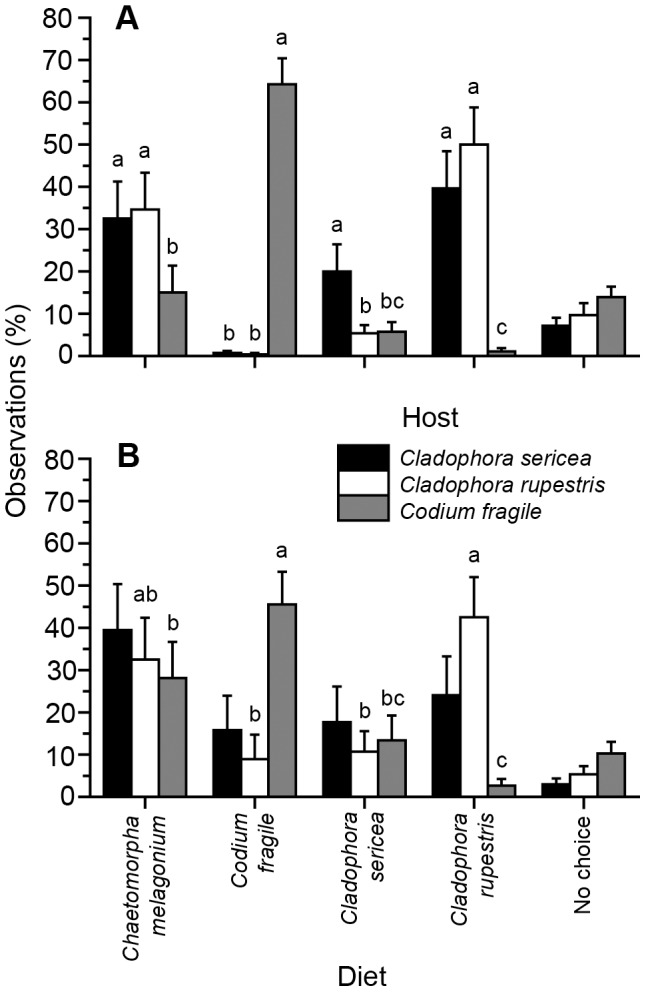
Observations (% of time; mean + SEM, n = 20 except small *Elysia viridis* from *Codium fragile* and *Cladophora sericea* where n = 16 and 19 respectively) of A) large and B) small *E. viridis* individuals from different original algal hosts (*C. sericea*, *Cladophora rupestris*, and *C. fragile*) on different algal diet species (*Chaetomorpha melagonium*, *C. fragile*, *C. sericea*, and *C. rupestris*) in 40 h multiple-choice assays. Different letters above bars within each original host species indicate significant differences at α = 0.05 (Friedman's PHC). The no choice category denotes observations where *E. viridis* were not on an algal diet species, these are not included in statistical analyses.

*E. viridis* from different original hosts demonstrated statistically significant preferences for different algal diet species (Friedman's test, large *E. viridis* from *C. sericea*: χ^2^ = 14.08, df = 3, *P* = 0.003; *C. rupestris*: χ^2^ = 25.00, df = 3, *P*<0.001; and *C. fragile*: χ^2^ = 36.99, df = 3, *P*<0.001; small *E. viridis* from *C. rupestris*: χ^2^ = 9.72, df = 3, *P* = 0.021; and *C. fragile*: χ^2^ = 20.29, df = 3, *P*<0.001), except for small *E. viridis* from *C. sericea* (Friedman's test, χ^2^ = 2.221, df = 3, *P* = 0.528). Large *E. viridis* from the two *Cladophora* hosts preferred the septate filamentous species *C. melagonium*, *C. sericea* and *C. rupestris* and were observed <1% of the time on the siphonous fleshy *C. fragile*, while large *E. viridis* collected from *C. fragile* showed a strong preference for *C. fragile* (Friedman's PHC, *P*<0.05; Fig. 1a). Small *E. viridis* collected from *C. rupestris* preferred *C. rupestris*, and small *E. viridis* from *C. fragile* preferred *C. fragile* (Friedman's PHC, *P*<0.05; Fig. 1b), although the preference was not as pronounced as for large slugs.

### *Elysia viridis* consumption and growth

**Figure 2:**
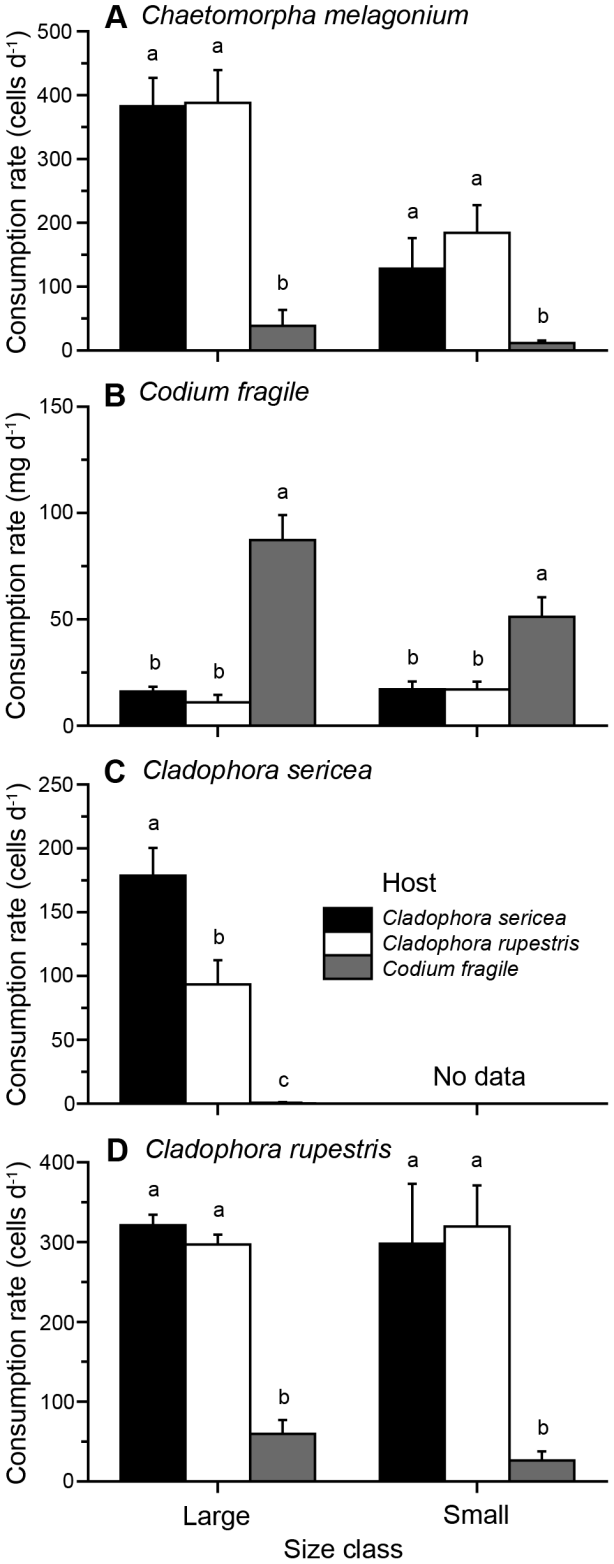
Consumption rate (cells or mg d^−1^; mean + SEM, n = 10) of large and small *Elysia viridis* individuals from different original algal hosts (*Cladophora sericea*, *Cladophora rupestris*, and *Codium fragile*) on different algal diet species A) *Chaetomorpha melagonium*, B) *C. fragile*, C) *C. sericea*, and D) *C. rupestris*. Different letters above bars within each *E. viridis* size class indicate significant differences at α = 0.05 (R-E-G-W *F* procedure).

Large and small *E. viridis* individuals collected from different original algal hosts demonstrated varying consumption on *C. melagonium* (ANOVA, large *E. viridis*: *F*_2, 27_ = 49.46, *P*<0.001; small *E. viridis*: *F*_2, 27_ = 15.23, *P*<0.001), *C. sericea* (ANOVA, large *E. viridis*: *F*_2, 27_ = 208.71, *P*<0.001; small *E. viridis*: data not analyzed as both treatment and control algal pieces became unhealthy and discolored), *C. rupestris* (ANOVA, *F*_2, 27_ = 99.135, *P*<0.001; small *E. viridis*: *F*_2, 27_ = 35.442, *P*<0.001), and *C. fragile* (ANOVA, large *E. viridis*: *F*_2, 27_ = 34.984, *P*<0.001; small *E. viridis*: *F*_2, 27_ = 6.84, *P* = 0.004). *E. viridis* individuals collected from both *Cladophora* hosts consumed more *C. melagonium, C. sericea*, and *C. rupestris* compared to *E. viridis* individuals collected from *C. fragile* (R-E-G-W *F*, *P*<0.05; Fig. 2a,c,d). In contrast, *E. viridis* individuals from *C. fragile* hosts consumed significantly more *C. fragile* compared to *E. viridis* from both *Cladophora* hosts (R-E-G-W *F*, *P*<0.05; Fig. 2b).

**Figure 3:**
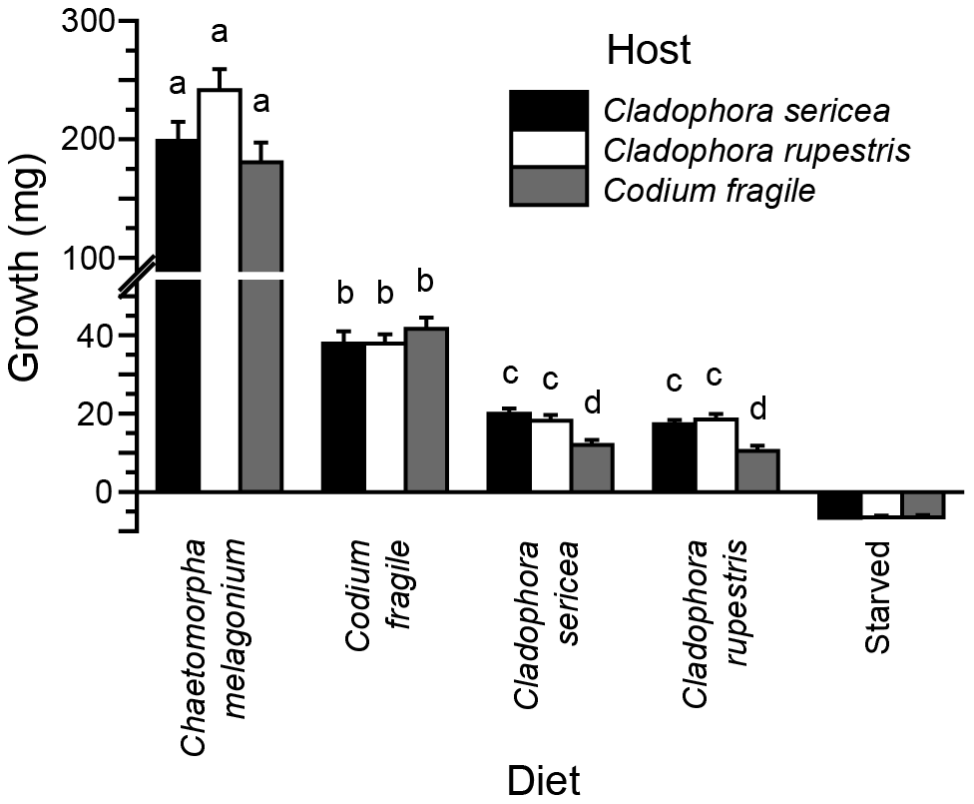
Growth (mg; mean + SEM, n = 20) of *Elysia viridis* individuals from different original algal hosts (*Cladophora sericea*, *Cladophora rupestris*, and *Codium fragile*) on different algal diet species (*Chaetomorpha melagonium*, *C. fragile*, *C. sericea*, and *C. rupestris*) or starved for 28 days. Different letters above bars indicate significant differences at α = 0.05 (R-E-G-W *F* procedure).

*E. viridis* individuals collected from different original algal hosts displayed varying growth on different algal diets species as shown by the statistically significant interaction between the two main factors (ANOVA, *F*_6,228_ = 2.60, *P* = 0.019). *E. viridis* from all hosts grew best on the non-host diet *C. melagonium*, followed by *C. fragile* and the two *Cladophora* diets, but *E. viridis* collected from *C. fragile* grew significantly less on the *Cladophora* diets compared to *E. viridis* from *Cladophora* spp. (R-E-G-W *F*, *P*<0.05; Fig. 3). Starved animals lost mass during the experiment (Fig. 3).

### Nutritional and morphological traits of algal diet species

**Figure 4:**
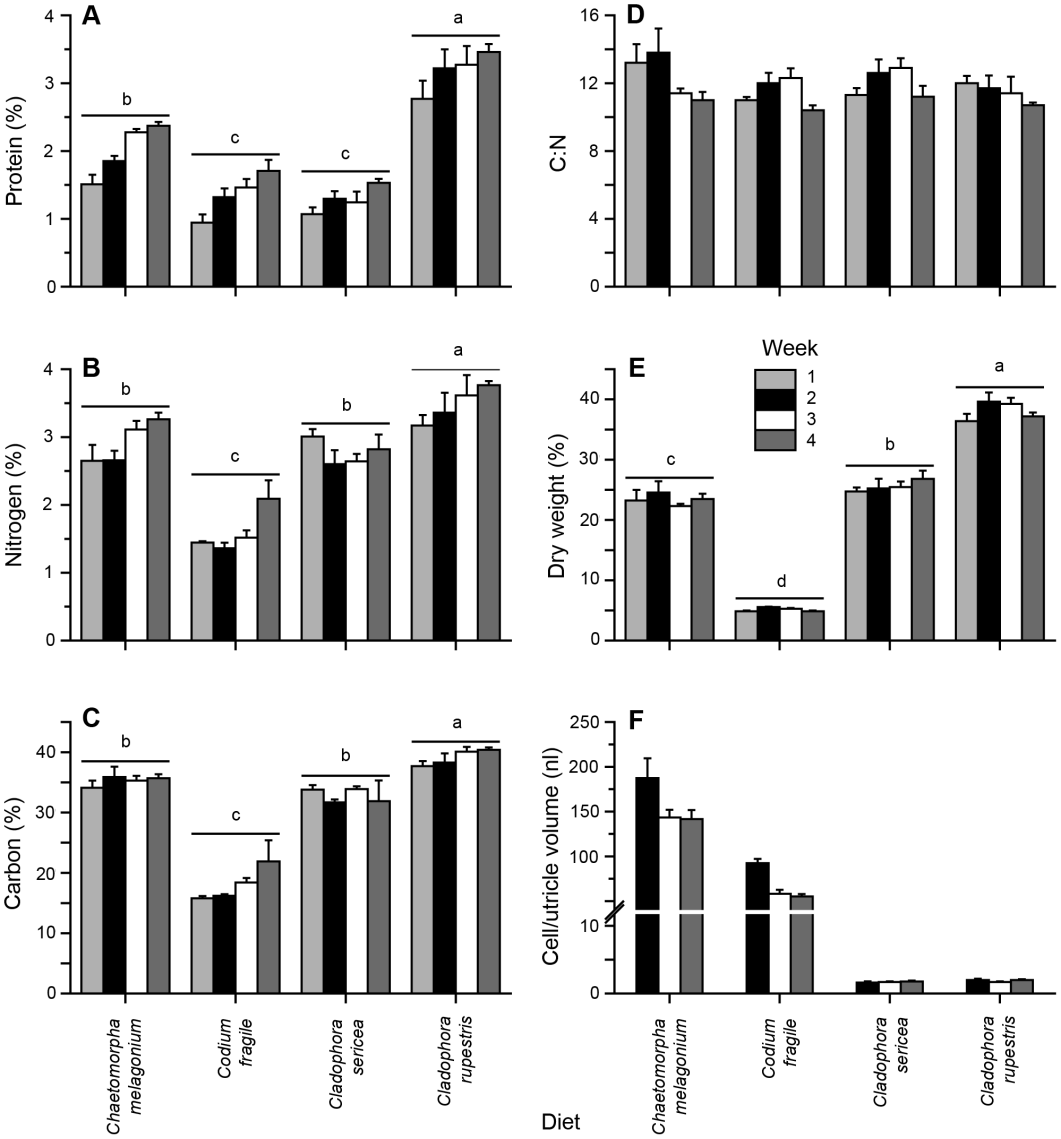
Tissue A) protein (% dry weight, mean + SEM, n = 3–5), B) nitrogen (% dry weight, mean + SEM, n = 5), and C) carbon content (% dry weight, mean + SEM, n = 5), D) C:N ratio, E) dry weight (% wet weight, mean + SEM, n = 5), and F) cell/utricle volume (nl, mean + SEM, n = 5) of the four algal diet species (*Chaetomorpha melagonium, Codium fragile, Cladophora rupestris* and *Cladophora sericea*) during the four week growth experiment with *Elysia viridis*. Different letters above bars indicate significant differences at α = 0.05 (R-E-G-W *F* procedure) between mean values for the different algal diet species.

All measured algal traits varied significantly between algal diet species and/or between collection weeks (Table 1). Nutritional traits (tissue P, N, and C content) showed fairly similar patterns between different algal diets (Fig. 4a–c). *C. rupestris* demonstrated the highest tissue P, N and C content followed by *C. melagonium*, *C. sericea* and *C. fragile* (R-E-G-W *F*, *P*<0.05; Fig. 4a–c). *C. fragile* consistently had the lowest nutritional content (Fig. 4a–c) although its' P content was similar to *C. sericea* (R-E-G-W *F*, *P*<0.05; Fig. 4a). There were no significant differences in the mean C:N ratio between algal diets (Table 1d, Fig. 4d). Percent dry weight followed a fairly similar pattern to algal nutritional traits with *C. rupestris* having the highest, *C. fragile* the lowest, and *C. sericea*, and *C. melagonium* intermediate values (Fig. 4e). Cell/utricle volumes of *C. melagonium* were on average 2.4 times larger than those of *C. fragile* across weeks (Fig. 4f), whereas *Cladophora* species had much smaller mean cell/utricle volumes that were fairly similar across sampling weeks (Fig. 4f). Decreases in mean cell/utricle volumes of approximately 25–40% from week 2 compared to weeks 3 and 4 occurred for both *C. melagonium* and *C. fragile* diets, which did not occur for the *Cladophora* diets (Fig. 4f), were the likely cause of the significant interaction term (Table 1f).

**Table 1:** Analysis of variance of A) protein content (% dry weight), B) nitrogen (N) content (% dry weight), C) carbon (C) content (% dry weight), D) C:N ratio, E) dry weight (% of wet weight), and F) cell/utricle volume (nl) of different algal diet species (A) in different weeks (W) during the growth experiment with *Elysia viridis*.

Source of variance	df	MS	*F*	p
**A) Protein**				
Algal diet species	3	15.39	124.81	**<0.001**
Week collected	3	1.56	12.69	**<0.001**
A×W	9	0.065	0.53	0.85
Residual	62	0.12		
**B) Nitrogen**				
Algal diet species	3	12.39	78.01	**<0.001**
Week collected	3	0.96	6.04	**0.001**
A×W	9	0.21	1.31	0.25
Residual	64	0.16		
**C) Carbon**				
Algal diet species	3	1693.42	154.96	**<0.001**
Week collected	3	22.05	2.02	0.12
A×W	9	12.28	1.12	0.36
Residual	64	10.93		
**D) C:N ratio**				
Algal diet species	3	4.04	1.69	0.18
Week collected	3	10.03	4.20	**0.009**
A×W	9	2.87	1.20	0.31
Residual	64	2.39		
**E) Dry weight**				
Algal diet species	3	2.92	1844.74	**<0.001**
Week collected	3	0.0034	2.13	0.11
A×W	9	0.0016	1.00	0.45
Residual	64	0.0016		
**F) Cell/utricle volume**				
Algal diet species	3	158.93	3003.30	**<0.001**
Week collected	2	0.44	8.38	**0.001**
A×W	6	0.25	4.66	**0.001**
Individual (A×W)	48	0.053	1.06	0.37
Residual	540	0.050		

There were no significant linear relationships between the nutritional content (i.e. P, N, C, as well as C:N ratio) or percent dry weight and *E. viridis* growth on the different algal diet species (Table 2). However, cell/utricle volume of the diet algal species was significantly correlated to the growth of *E. viridis* (growth  = 1.186 cell/utricle volume +1.439, Table 2).

**Table 2:** Simple linear regressions of mean *Elysia viridis* growth (mg) versus the mean protein content (% dry weight), nitrogen (N) content (% dry weight), carbon (C) content (% dry weight), C:N ratio, dry weight (% of wet weight), and cell/utricle volume (nl) of different algal diet species in different weeks during the growth experiment.

Growth versus	df	MS	*F*	P	R^2^
Protein (Residual)	1 (2)	0.80 (12772.12)	<0.01	0.99	<0.001
Nitrogen (Residual)	1 (2)	179.60 (12682.73)	0.01	0.92	0.007
Carbon (Residual)	1 (2)	799.70 (12372.68)	0.07	0.82	0.031
C:N (Residual)	1 (2)	13931.00 (5807.03)	2.40	0.26	0.545
Dry weight (Residual)	1 (2)	226.99 (12659.03)	0.02	0.91	0.009
Cell/utricle volume (Residual)	1 (2)	22970.83 (1287.11)	17.85	**0.05**	0.899

## Discussion

We found that the polyphagous marine herbivore *E. viridis* tended to reside on and consume algal species similar to their original algal host both in multiple-choice preference and no-choice consumption experiments in the laboratory. In general, both small and large slugs collected from *C. fragile* preferred and consumed *C. fragile*, while individuals collected from the *Cladophora* species preferred and consumed the *Cladophora* species and *C. melagonium*. The few previous studies investigating individual dietary specialization in marine herbivores by repeated observations of the same individual have found varying results. One example of inter-individual diet specialisation in sympatric conspecific sacoglossans is that of *Placida dendritica* [32]. Despite feeding on several macroalgal species at the population level, individual *P. dendritica* demonstrated an extreme limitation to switching from their original host to other species and generally died in the presence of non-preferred species that conspecific individuals consume [32]. In contrast, there was no variation in preference for three host algae of varying quality among individual herbivorous amphipods (*Peramphitoe parmerong*, [33]). Diet choice was strongly influenced by past diet, but individual amphipods selected different hosts to that on which they had recently been feeding [33]. In addition, feeding preferences of the isopod *Dynamene bidentata* were also influenced by previous dietary experience, but in this case individuals preferred the recently consumed species and showed a diet preference only after sampling the environment for different algal species [34]. Furthermore, host preference depended on original habitat type in the isopod *Idotea baltica*, where isopods from angiosperm assemblages occurred more often on the brown alga *Fucus vesiculosus* compared to individuals from algal assemblages [35].

Jensen [23] suggested that short-term switching to new hosts by *E. viridis* could be limited by the slugs' ability to recognize and/or handle algae from different functional groups. *E. viridis* feeds by piercing and sucking the cytoplasm from algal cells, and differences in the cell wall chemistry of the algal diets or the tooth morphology of the slugs could potentially constrain *E. viridis* switching to new hosts [23]. Previous studies have found variation in tooth morphology in different populations of *E. viridis* residing on different algal species [23], [36]. Slugs from Danish populations residing on *Chaetomorpha linum* possessed narrower and shorter teeth compared to French and English slugs found on *C. fragile* [23], [36]. However, whether this variation was due to different diets or genetic differences between different slug populations is not clear. Furthermore, Irish populations of *E. viridis* from different sympatric algal hosts demonstrated little variation in tooth shape [25]. We do not know if, and in that case how, the tooth morphology of Swedish *E. viridis* varies between individuals found on different algal hosts. In this study, *E. viridis* tended to choose and consume algal diet species with similar morphology (i.e. filamentous/septate vs. siphonous/coenocytic) and cell wall chemistry (cellulose in *C. sericea, C. rupestris*, and *C. melagonium* vs. mannan in *C. fragile*) [37], [38] to their original algal hosts. The cell volume of the *Cladophora* species was much smaller than that of *C. fragile* and if *E. viridis* from *C. fragile* possess teeth that are too large to feed on *C. sericea* and *C. rupestris* it could prevent efficient consumption. However, as the slug growth was independent of original algal host, tooth morphology likely has little effect on Swedish *E. viridis*'s ability to feed on different algal diets in the long term.

There was a clear and pronounced difference in long-term growth when *E. viridis* were offered different algal diet species in the present study. However, the algal species preferred and consumed in the short-term experiments were not the species that had the highest fitness value in terms of growth, as all slugs grew best when fed the non-host *C. melagonium*. *E. viridis* individuals offered *C. melagonium* were approximately 5 times larger compared to conspecifics offered the next best diet *C. fragile*, and about 10 times larger than slugs offered any of the *Cladophora* species in the end of the experiment. Furthermore, the growth of *E. viridis* was not correlated to any of the measured nutritional algal traits (tissue P, N or C content) or to percent dry weight, but did correlate positively to the cell/utricle volume of the algal species. Because *E. viridis* is a suctorial feeder, the large-celled *C. melagonium* and *C. fragile* likely provide the slugs with more cytoplasm per unit feeding effort and a greater amount of energy per unit time. One alternative explanation for these results is that the growth value of different algal diets was affected by the ability of *E. viridis* to retain functional chloroplasts from its algal food (kleptoplasty). However, although kleptoplasty has a demonstrated advantage to sacoglossan survival during periods of food shortage (e.g. [19], [39]), and kleptoplasts may play a role in nitrogen acquisition for *E. viridis* [40], the contribution of photosynthates to sacoglossan energy budgets through kleptoplasty is still poorly defined [41]. Furthermore, in this study light was kept low in order to reduce possible somatic growth derived from photosynthetic kleptoplasts, and should not have affected the long-term growth of *E. viridis*.

It is clear from the results presented in the present study that the energy assimilation per unit time alone does not explain host selection of individual *E. viridis* as predicted by the ODT. Furthermore, if host choice were based solely on the energetic value of the algal diet, *C. melagonium* and *C. fragile* hosts would house a disproportionately greater number of *E. viridis* compared to *Cladophora* hosts in natural slug populations. However, field studies conducted in the study area show that *E. viridis* occurred in greater abundance on sympatric *C. sericea* and *C. rupestris* hosts compared to *C. fragile* hosts and were totally absent from *C. melagonium* [26]. Moreover, *E. viridis* individuals on *C. rupestris* hosts were larger compared to individuals on *C. fragile* [26]. This suggests that, in the field, variables other than energy maximisation (e.g. shelter from wave action or predation) [42] are important for determining the suitability of host algae to *E. viridis*. For example, predation can have a strong impact on habitat use and foraging behavior in a variety of animals, causing a forager to select sub-optimal diets or hosts to either stave off or avoid predators (e.g. [43], [44]). Furthermore, for small herbivores that reside on the plants they consume, food and habitat are closely tied so the refuge value of a host may be as important as its dietary value in determining patterns of diet selection [42], [45], [46]. We are not aware of any published reports of predators that use *E. viridis* as prey, but several potential predators (e.g. different species of crabs and fish) co-occur with *E. viridis* in the study area (G. B. Toth, pers. obs.). Therefore, predation could be a potential explanation for the discrepancy between the growth values of different algal diet species found in this study, and patterns of abundance and size distribution on different algal species in natural *E. viridis* populations [26].

In conclusion, our study shows high within-population variation in feeding and host preference in *E. viridis*. Slug individuals collected from different algal hosts in the field tend to choose and consume hosts with similar morphology and cell wall chemistry in short-term laboratory experiments, irrespective of the growth value of the algal diets. Together, the results presented in the present study indicate that host choice in *E. viridis* is not determined by the energetic value of seaweed diets as predicted by the ODT, but that factors other than nutrition (e.g. predation) are important for host/diet selection in this species. Since many small marine herbivores are considered broad generalists, they may constitute a suitable group of model organisms for studies on individual specialization at lower trophic levels.
